# Understanding Microbial Loads in Wastewater Treatment Works as Source Water for Water Reuse

**DOI:** 10.3390/w13111452

**Published:** 2021-05-21

**Authors:** Hodon Ryu, Yao Addor, Nichole E. Brinkman, Michael W. Ware, Laura Boczek, Jill Hoelle, Jatin H. Mistry, Scott P. Keely, Eric N. Villegas

**Affiliations:** 1 Center for Environmental Solutions and Emergency Response, Office of Research and Development, United States Environmental Protection Agency, Cincinnati, OH 45268, USA;; 2 Center for Environmental Measurement and Modeling, Office of Research and Development, United States Environmental Protection Agency, Cincinnati, OH 45268, USA;; 3 United States Environmental Protection Agency, Region 6, Dallas, TX 75270, USA;

**Keywords:** water reuse, microbial loads, waterborne pathogens, indicators

## Abstract

Facing challenges in water demands and population size, particularly in the water-scarce regions in the United States, the reuse of treated municipal wastewater has become a viable potential to relieve the ever-increasing demands of providing water for (non-)potable use. The objectives of this study were to assess microbial quality of reclaimed water and to investigate treatability of microorganisms during different treatment processes. Raw and final treated effluent samples from three participating utilities were collected monthly for 16 months and analyzed for various microbial pathogens and fecal indicator organisms. Results revealed that the detectable levels of microbial pathogens tested were observed in the treated effluent samples from all participating utilities. Log_10_ reduction values (LRVs) of *Cryptosporidium* oocysts and *Giardia* cysts were at least two orders of magnitude lower than those of human adenovirus and all fecal indicator organisms except for aerobic endospores, which showed the lowest LRVs. The relatively higher LRV of the indicator organisms such as bacteriophages suggested that these microorganisms are not good candidates of viral indicators of human adenovirus during wastewater treatment processes. Overall, this study will assist municipalities considering the use of wastewater effluent as another source of drinking water by providing important data on the prevalence, occurrence, and reduction of waterborne pathogens in wastewater. More importantly, the results from this study will aid in building a richer microbial occurrence database that can be used towards evaluating reuse guidelines and disinfection practices for water reuse practices.

## 1. Introduction

Water scarcity is becoming a global problem directly impacting drinking water quality and quantity throughout the US, especially in regions with limited source waters. In recent years the United States (US) has been experiencing longer, more frequent, and more severe drought periods, especially in the southwestern regions [1]. To address this challenge of continued high demand for high-quality water, especially for potable use, many communities, water resource managers, and government agencies in the US and worldwide are turning to various alternative solutions that are more sustainable, cost-effective, and responsive to changing community demands and climate fluctuations [2,3]. Diminishing freshwater supplies and increasing municipal water demands in highly populated arid areas make water reuse a feasible alternative [4]. The reuse of treated municipal wastewater has the potential not only to relieve the ever-increasing demands of providing fresh water, but also to provide several beneficial purposes. Water reuse is well established in the US [5], and it is now recognized as an important integral component of water resources management particularly in arid regions of the world [4,6].

There are three types of water reuse scenarios: (1) de facto water reuse, (2) non-potable, and (3) direct and indirect potable. De facto reuse is the use of discharged treated wastewater upstream of waterbodies used as a source for drinking water, which has been occurring for many years [7]. Historically, reclaimed water (i.e., treated wastewater in reuse for beneficial purpose) was first used for non-potable applications such as in agriculture, which do not require high water quality standards and even are often perceived as a method for wastewater disposal [8]. In the last several decades, the trend of applications of water reuse has shifted toward higher level uses such as indirect potable reuse (IPR) practices that are applied to recharge ground water sources, urban irrigation, toilet and urinal flushing, commercial and industrial uses, and finally, potable reuse [5]. More recently, direct potable reuse (DPR) has already been implemented in some communities and seriously considered in others to solve the water scarcity issues [9–13]. As the consideration of DPR is expected to increase, a large portion of the population will rely on treated wastewater as a drinking water source. However, negative public perception and potential health concerns due to the uncertainty of the quality of water for reuse purposes have limited its widespread adoption. Moreover, there are practical issues associated with treatment engineering: (1) an operator with an advanced treatment license is needed to operate the plant, which can be costly many municipalities and (2) once the drought situation ends, the dependence on DPR practices is reduced and thus scaled back for economic reasons.

Breakdowns or overloading of microbial pathogens in municipal water utilities have resulted in community outbreaks of gastroenteritis [14]. From a microbiological perspective, questions arise about density levels of pathogens and tools for microbial risk assessment. While many studies have reported the occurrence of enteric microbial pathogens in non-potable reuse water and their associated risks [15–18], there has been a dearth of information on microbial surveillance in DPR-associated water usage. More recently, a quantitative microbial risk assessment (QMRA) was proposed as a useful tool for predicting risks associated with various DPR scenarios [19–22]. However, actual microbial densities and the assessment of reduction values obtained within various wastewater treatment processes remain limited.

With the emergence of technology that makes wastewater effluent a viable alternative source of potable water, additional research is needed to further characterize the microbial pathogens of concern present in effluents and the risks associated with exposure to these contaminants as they relate to DPR practices. To date, while there have been several guidance documents that have been published, there are still no federal regulations that ensure the adoption and implementation of DPR processes that are protective of public health. Because of the lack of such regulations, individual states in the US (Arizona, California, Colorado, Florida, Texas, and Washington) are establishing their own standards (https://watereuse.org/advocacy/state-policy-and-regulations, accessed on 20 May 2021). In this study, we attempted to address the efficacies of treatment processes typically employed in municipal wastewater treatment plants, focusing on the removal or inactivation of waterborne pathogens (e.g., bacteria, viruses, and parasites) from the influent to the effluent. The data gathered provide the much-needed information to assist policy makers to develop better guidance on the safe use and application of DPR practices throughout the US.

## 2. Materials and Methods

### 2.1. Sample Collection and Processing

Wastewater samples from three anonymized wastewater treatment plants in Texas were collected monthly for 16 months between March 2015 and June 2016. Treatment characteristics and processes used by the participating utilities are listed in Table 1. Two sampling points in each plant, such as influent (raw) and effluent (final treated) sites, were selected (Table S1). For each sampling event, 1 L influent (raw) and effluent (final, post-disinfection) grab samples were taken and placed into sterile bottles. In addition, a 100 L effluent sample was filtered on site using a hollow fiber ultrafilter (HFUF; Rexeed-25S, Asahi Kasei Kuraray Medical Co. Ltd., Tokyo, Japan) as previously described [23]. All samples were immediately shipped overnight on wet ice to US Environmental Protection Agency (USEPA), Cincinnati, Ohio. Samples were processed immediately upon receipt. A sampling, processing, and analysis scheme is shown in Figure S1.

Additional details about sample volumes collected and the amount analyzed for microorganisms tested are presented in Table S1. Briefly, the 1 L influent grab samples were aliquoted for bacterial, viral, and protozoan analyses. Five 100 mL aliquots were taken for total coliform, fecal coliform, *Escherichia coli*, endospore, and protozoan analyses, and the remaining sample volume was taken for viral analyses (10 mL and 400 mL aliquots for bacteriophage and human adenovirus, respectively). The 1 L effluent grab sample was divided into one 10 mL and four 100 mL aliquots for bacteriophages and bacterial indicator analyses, respectively.

One hundred liters of effluent samples was concentrated as described above. The retentate was eluted with 400 mL of elution solution and the eluate was adjusted to pH 7.0–7.5 as previously described [23]. One 10 mL aliquot was transferred into a sterile 15 mL conical tube and stored at 4 °C for bacteriophage analysis. The remaining eluate was then carefully decanted into two 250 mL conical tubes and centrifuged at 1500× *g* for 15 min with no brake at 4 °C. The pellets were then analyzed for *Giardia* and *Cryptosporidium* as per USEPA Method 1623.1 [24,25]. The remaining supernatant was transferred into a 50 mL conical tubes and stored in −80 °C for adenovirus analysis.

### 2.2. Microbial Analyses

Microorganisms tested included waterborne bacteria (total and fecal coliforms, *Escherichia coli*, and aerobic endospores), protozoan parasites (*Cryptosporidium* oocysts and *Giardia* cysts), and viruses (male-specific and somatic bacteriophages and human adenovirus). Standard methods, USEPA methods, and a newly developed viral infectivity assay were used for the detection of the respective test microorganisms as described below.

#### 2.2.1. Fecal Indicator Organisms

Total coliforms and *E. coli* were assessed using IDEXX Colilert Quanti-Tray System as per the manufacturer’s procedures (https://www.idexx.com/files/colilert-procedure-en.pdf, accessed on 20 May 2021). The most probable numbers (MPN) calculator was used to determine the concentrations of these bacteria as appropriate [26]. USEPA Method 1681 [27] was used to measure fecal coliforms in all samples analyzed in this study, and aerobic endospores were assessed following a standard methods procedure [28]. Male-specific and somatic bacteriophage concentrations were measured using USEPA Method 1602 [29].

#### 2.2.2. Protozoan Parasites

Samples collected from influent and effluent samples were analyzed for *Cryptosporidium* oocysts and *Giardia* cysts by immunomagnetic separation (IMS) followed by immunofluorescence assay (IFA) as described in Method 1623.1 [24]. Briefly, 0.5 mL packed pellets equivalents from concentrated samples were transferred to a 15 mL conical tube. The sample tube was rinsed twice with 1 mL of 5% sodium hexametaphosphate (NaHMP;Sigma, St. Louis, MO, USA), and the final volume was brought to 8 mL with sterile water. The samples were then processed as described in Method 1623.1 with the following modifications: Leighton tubes and magnets were replaced by 15 mL conical tubes, DynaMag 15, and DynaMag 2; magnetic materials were removed as described in the Method (Section 13.4.1) [24], except the tubes were rinsed with 1 mL of 5% NaHMP and 1 mL of 10% BSA in PBS; an additional 10 mL of PBS wash was performed after IMS (Section 13.4.3), and a single heat dissociation was used [24,30]. Samples were stained using EasyStain^™^ G/C (biopoint, Pittsburg, PA, USA) following the manufacturer’s instructions except the wash and fixation steps were omitted. Cyst and oocyst enumeration was done microscopically as per Method 1623.1, except only the first 20 of the antibody-stained (oo)cysts were analyzed by 4′,6′-diamidino-2-phenylindole (DAPI) staining. One IMS reaction was performed per sample.

#### 2.2.3. Adenovirus Infectivity Assay

##### Adenovirus and mammalian cell line propagation.

Human adenovirus type 2 (AdV2) was obtained from the American Type Tissue Collection (ATCC^®^ VR-846, Manassas, VA, USA). A549 cells, a human lung carcinoma, were obtained from ATCC (ATCC^®^ CCL-185) and seeded into 175 cm^2^ vented tissue culture flasks (Greiner, Monroe, NC, USA) in Dulbecco’s minimum essential medium (DMEM) (Thermo Fisher Scientific Inc., Waltham, MA, USA) supplemented with 10% fetal calf serum (FCS; Thermo Fisher Scientific Inc.), and maintained in 5% (vol/vol) CO_2_. AdV2 was propagated in A549 cell cultures. Briefly, A549 cells were inoculated with stock AdV2, and the cells were incubated for up to two weeks or until cytopathic effects (CPE) were observed. Cells underwent three freeze-thaw cycles, and a portion of the cell lysate was passaged onto fresh A549 cells for further propagation. Cell lysates from all passages were combined to obtain maximal viral stock. Lysates were centrifuged at 2500× *g* for 30 min to remove cell debris. The supernatant-containing virus was then centrifuged at 10,000× *g* for 10 min to remove any remaining small cellular debris. In order to remove any potential viral clumps, remove any remnants of growth media, and further purify the viral stock, the virus supernatant was further concentrated using celite, as described by McMinn et al. [31]. Following celite concentration, viral stocks were further concentrated using 30 kDa MWCO Vivaspin-20 ultrafilters (Sartorius-Stedim, Aubagne, France) as described previously [32]. This resulted in stock titers of 10^10^ MPN/mL, which were then stored at −80 °C.

##### Cell culture analysis.

AdV2 titers from all samples were determined using a total culturable virus assay (TCVA) with A549 cells. Test cultures for TCVA were grown in round-bottom cell culture tubes (BD Falcon, Franklin Lakes, NJ, USA) in the same media as described above, but supplemented with an antibiotic-antimycotic solution (Invitrogen, Grand Island, NY, USA) and incubated in ambient air at 37 °C. Test cultures were inoculated 5 days after planting. Prior to all infections, A549 cells were washed with 2 mL of Earle’s balanced salts solution. Each sample and viral seed underwent five-fold dilutions in PBS. Then, 4 dilution series from each sample were divided among 5 replicate A549 cell culture tubes (5 tubes per dilution). Next, 200 μL of inoculum was introduced to each tube, and all tubes were placed into a roller apparatus at 37 °C for a minimum of 90 min before maintenance media consisting of DMEM (Hyclone) with 2% FCS and antibiotic-antimycotic liquid (Invitrogen) were added. All cultures were incubated for 21 days at 37 °C and were checked weekly for cytopathic effects (CPE). Those tubes which exhibited 75%–100% CPE were immediately marked as positive. A most probable number (MPN) approach was used to estimate the number of infectious units in each sample based on the number of positive replicates in the five-fold dilution series for each of the samples. The MPN software used can be found at https://mostprobablenumbercalculator.epa.gov (accessed on 20 May 2021). The MPN values were then log_10_ transformed for further data analyses.

##### Integrated cell culture quantitative PCR (ICC-qPCR).

Infectious virus concentrations were determined using ICC-qPCR as previously described [33]. Briefly, test cultures for ICC-qPCR were grown in 24-well cell culture plates (Thermo Fisher Scientific Inc., Waltham, MA) in the same media as described above. Sample inoculation procedures were followed as described above for TCVA, with the exception of 100 μL of inoculum that was added to three wells per dilution. To develop an ICC-qPCR standard curve, five 10-fold serial dilutions from virus stock (i.e., 10^5^–10 MPN/100 μL) were inoculated into three wells for each dilution of series. The trays were then incubated for 48 h at 37 °C in a 5% CO_2_ incubator. After incubation, the cell monolayers were washed five times with 1 mL of PBS to remove non-infectious extracellular viruses and then harvested by using a combination of freeze-thaw cycles and the scraping method. Note that it is possible that the wash steps did not completely remove all the non-infectious virus attached to cells, which could result in a false positive reading. To overcome this, four rinse control replicates with heat-inactivated adenoviruses were processed along with each set of ICC-qPCR standards as described by Gerrity et al. [34]. Viral DNA from cell harvest with infectious viruses was extracted and purified with the DNeasy 96 blood and tissue kit (QIAGEN, Valencia, CA, USA) according to the manufacturer’s instructions. DNA extracts were stored at −20 °C until the qPCR assay.

The qPCR assay was performed in 25 μL reaction mixtures containing 1X TaqMan universal PCR master mix with AmpErase uracil-N-glycosylase (Applied Biosystems, Foster City, CA, USA), 0.2 μM (final concentration) of each primer, and a VIC-labeled hydrolysis probe. The forward and reverse primers (AdF1B, 5′-GGAYGCYTCGGAGTACCTGAG-3′, and AdR2B, 5′-ACSGTGGGGTTYCTAAACTTGTT-3′) and hydrolysis probe (AdP1M, 5′-VIC-TGGTGCAGTTYGCC-MGBNFQ-3′) were designed for the detection of human adenovirus including type 2 using primer express software (Applied Biosystems, Foster City, CA, USA). The amplification protocol involved an initial incubation at 50 °C for 2 min to activate uracil-N-glycosylase, followed by 10 min of incubation at 95 °C to activate AmpliTaq Gold^™^ enzyme, and then 40 cycles of 95 °C for 15 s and 60 °C for 1 min. The qPCR assays were performed using a 7900 HT Fast Real-Time Sequence Detector (Applied Biosystems). All assays were performed in triplicate in MicroAmp Optical 96-well reaction plates with MicroAmp Optical Caps (Applied Biosystems, Foster City, CA, USA). PCR data were analyzed using ABI’s sequence detector software (version 2.2.2). Two independent standard curves for each qPCR assay were generated by plotting the threshold cycle (CT) values against the number of target copies corresponding to serially diluted plasmid standards purchased from IDT-integrated DNA technologies (Coralville, Iowa, USA). The target copy numbers (T) were estimated by the following equation

(1) $$\text{T}=\left[\text{D}/\left(\text{PL}\times 660\right)\right]\times 6.022\times 10^{23}$$

where D (g/μL) is plasmid DNA concentration and PL (bp) is plasmid length in base pairs. Each standard curve was generated from at least five 10-fold plasmid dilutions in triplicates. Two no-template controls per PCR plate were used to check for cross-contamination.

The ICC-qPCR standard curve was constructed using five 10-fold serial dilutions of the AdV2 stock as described above. Briefly, based on the quantities resulting from the ICC-qPCR assay (x) and the original number of infectious AdV2 spiked into each dilution (y), the regression analysis was performed for determining unknown parameter (A) in the standard curve (i.e., y = Ax). The concentrations of infectious AdV2 in preinoculation samples (i.e., the dependent variable, y = the original virus concentration prior to replication) were estimated using the regression equation with ICC-qPCR quantities (i.e., the independent variable, x). The estimated y values were used to calculate the log_10_ inactivation of AdV2.

### 2.3. Data Presentation and Statistical Analyses

Log_10_ reduction (LR) was defined by the following equation:

(2) $$\text{LR}=-{\text{log}}_{10}\left({\text{N}}_{\text{e}}/{\text{N}}_{\text{i}}\right)$$

where N_i_ and N_e_ are the concentrations of culturable/infectious microorganisms in influent and effluent samples, respectively. All non-detects were assigned the value of the detection limits (DLs).

Data collected were compiled and organized using MS Excel (Microsoft, Redmond, WA, USA). Data exploration, analysis, and visualization were performed using SigmaPlot^®^ 13 and Sigma Stat (Systat, San Jose, CA, USA), GraphPad (GraphPad Software, San Diego, CA, USA), R and RStudio (R Studio, Boston, MA, USA), and/or PAST 3.2 (http://priede.bf.lu.lv/ftp/pub/TIS/datu_analiize/PAST/2.17c/download.html, accessed on 20 May 2021) statistical analysis softwares.

## 3. Results and Discussion

Microbial pathogens such as *Cryptosporidium, Giardia*, and infectious human adenovirus were monitored from a total of 47 influents (raw) and 47 final treated effluent samples. A total of 45 samples for each of the sampling points were analyzed for microbial indicators such as male-specific coliphages, somatic coliphages, total coliforms, fecal coliforms, *E. coli*, and aerobic endospores. Microbial concentrations before and after treatment were assessed, and LRVs were calculated and described for the three participating plants.

### 3.1. Enteric Protozoan Parasites

*Giardia* cysts were detected in 98% (46/47) and 96% (45/47) of influent and effluent samples, respectively, whereas 66% (31/47) and 83% (39/47) were positive for *Cryptosporidium* oocysts (Table 2), respectively. Compared to the effluent sample results, there was a lower prevalence of *Cryptosporidium* in influent samples. In the present study, sample volumes were constant with the collected volumes of influent and effluent samples being about 100–125 mL and 100 L, respectively (Table S1). However, various equivalent sample volume analyzed (ESVA) among samples were observed, and this variance was mainly caused by differences in the IMS processing volume that is directly related to the amount of solids in the water samples (i.e., changes in water turbidity). IMS capacity limitations may reduce the actual volume of the sample analyzed. The ESVA was about 100 mL of influent grab samples and a range of 10–100 L of effluents. Accordingly, for the latter, detection limits (DLs) ranged from 0.1 to 0.01 (oo)cysts/L. The lower DLs of effluent samples can explain the higher prevalence with lower mean concentrations of (oo)cysts than influent raw samples with a DL of 8–10 (oo)cysts/L.

Box and whisker plot analyses of the levels of *Cryptosporidium* oocysts (Figure S2) and *Giardia* cysts (Figure S3) in the three wastewater treatment plants were also performed, and the statistics are presented in Table 2. Numerous studies have reported microbial concentrations of various waterborne pathogens in raw and treated wastewater. The concentrations of protozoan parasites in raw wastewater ranged between 0.3 and 5.0 × 10^4^ oocysts/L and 3.2 and 9.0 × 10^4^ cysts/L for *Cryptosporidium* spp. and *Giardia* spp., respectively [35–40]. In the present study, *Giardia* cysts were detected more often and at higher concentrations than *Cryptosporidium* oocysts in both influent and effluents samples. This result is consistent with other reported studies [17,41,42]. Among the three participating plants, approximately one order of magnitude greater mean concentrations of *Cryptosporidium* and *Giardia* with 693 and 4492 (oo)cyst/L were detected in influent samples from the Plant 2. In effluent samples, Plant 2 also had the highest mean concentration of *Cryptosporidium* at 4.1 oocyst/L, whereas *Giardia* showed no marked differences among the three plants. Indeed, several studies including the present study reported higher reduction of *Giardia* than *Cryptosporidium* throughout the wastewater treatment processes. Taran-Benshoshan et al. [42] reported a higher removal of *Giardia* cysts by secondary and tertiary treatment processes, resulting in lower cyst concentrations in treated effluents than *Cryptosporidium* oocysts. By contrast, some studies reported higher concentrations of *Cryptosporidium* than those of *Giardia* in treated effluents [41,43]. One possible explanation of this discrepancy is relatively high levels of *Cryptosporidium* in influent raw water.

### 3.2. Human Adenovirus

Infectious human adenoviruses were measured throughout the 16-month sampling period, and their prevalence and concentrations were presented in Table 2. The prevalence of infectious adenovirus markedly decreased to 60% (28/47) in treated effluent samples, whereas all the influent samples except for one sample from Plant 3 were positive (98%; 46/47). Box and whisker plot analyses were performed in order to visualize the range of concentrations of human adenovirus (Figure S4). The reported adenovirus concentrations in raw wastewater varied between 56 and 6900 infectious units (IU)/L [44–46]. In influent samples, Plant 1 showed a broad range of viral concentrations with the highest median concentration of about 3000 MPN/L, followed by Plant 2 and Plant 3 with median values of about 60 and 20 MPN/L, respectively. Relatively low concentrations of infectious viruses were detected in most effluent samples with a range of 0.02–0.5 MPN/L. Higher levels of AdV were detected; however, in three out of nine effluent samples from Plant 1. These higher AdV concentration levels could be due to the population density being served by the plant. Nevertheless, the higher influent concentrations allowed for a much wider dynamic range for determining LRVs for this particular plant, especially with regards to UV treatment efficacies.

### 3.3. Microbial Indicators

Bacteriophages were detected in all 1 mL influent grab samples, whereas 2 and 1 of the 45 1 mL effluent grab samples were positive for male-specific and somatic bacteriophages, respectively (Table 3). The calculated DL for the 1 mL grab sample was 1 PFU/mL. In order to increase ESVA (i.e., acquire lower DLs), 100 L effluent samples were filtered by HFUF, and 1 mL of 400 mL filter eluates were analyzed for bacteriophages. The resulting ESVA of effluent-HFUF was increased to 250 mL with a DL of 0.004 PFU/mL. The increased ESVA and DL resulted in a relatively high prevalence of bacteriophages with 11% (5/45) and 20% (9/45) of effluent-HFUF samples being positive for male-specific and somatic bacteriophages, respectively, versus 2% (1/45) and 4% (2/45), respectively, for effluent grab samples. As expected, most positive effluent-HFUF samples showed relatively low levels of bacteriophages, which are at least three orders of magnitude lower than the DL of 1 PFU/mL for effluent grab samples (Table 3).

Whereas a vast majority of effluent samples were below detection limits, results revealed relatively high levels of bacteriophages in the raw influent samples with marked reductions in concentrations detected in the effluent samples (Figure S5). The highest median concentrations of both bacteriophages were observed in influent samples from Plant 2. Compared to infectious human adenovirus, concentrations of bacteriophages in raw influent samples were at least three orders of magnitude higher. By contrast, relatively low prevalence with much lower concentrations was observed for both indicators in the effluent samples. Bacteriophages are usually applied as a viral indicator of human pathogenic viruses while monitoring microbial quality of environmental water. The relatively high concentrations of bacteriophages in raw wastewater further highlight their potential use as conservative indicators of infectious human adenoviruses when assessing the presence of human pathogenic viruses in raw wastewater. In general, human viruses are highly infectious (i.e., only requiring one virus particle to cause disease in humans) and are found in very low concentrations, thus making them difficult to detect in environmental water.

The bacterial indicators tested were detected in all the influent grab samples, and their levels were relatively high (Table 4). Whereas aerobic endospores and total coliforms were detected in all effluent grab samples, only 56% (25/45) and 69% (31/45) of the samples were positive for *E. coli* and fecal coliforms, respectively. For the latter two bacteria, the highest prevalence was observed in Plant 1 (100%), followed by Plant 2 and Plant 3 (Table 4). The ESVA was 100 mL of influent and effluent grab samples with a DL of 1 CFU/100 mL. Levels of *E. coli* (5.00–8.84 CFU/100mL), fecal coliforms (5.00–9.38 CFU/100 mL), and total coliforms (5.30–9.37 CFU/100 mL) were relatively high in influent samples, while low to below detection levels were found in many of the effluent samples (0.30–2.81 CFU/100 mL) (Table 4). Concentrations of aerobic endospores in influent samples were more than three orders of magnitude lower than those of the other bacterial indicators. In addition, aerobic endospores were detected in all the effluent samples with relatively high concentrations, demonstrating their relative resistance to wastewater treatment processes including disinfection practices (e.g., chlorine and UV) evaluated in this study [47].

### 3.4. Calculated Log_10_ Reduction Values (LRVs) Obtained from the Three Wastewater Treatment Plants

**Figure 1.**
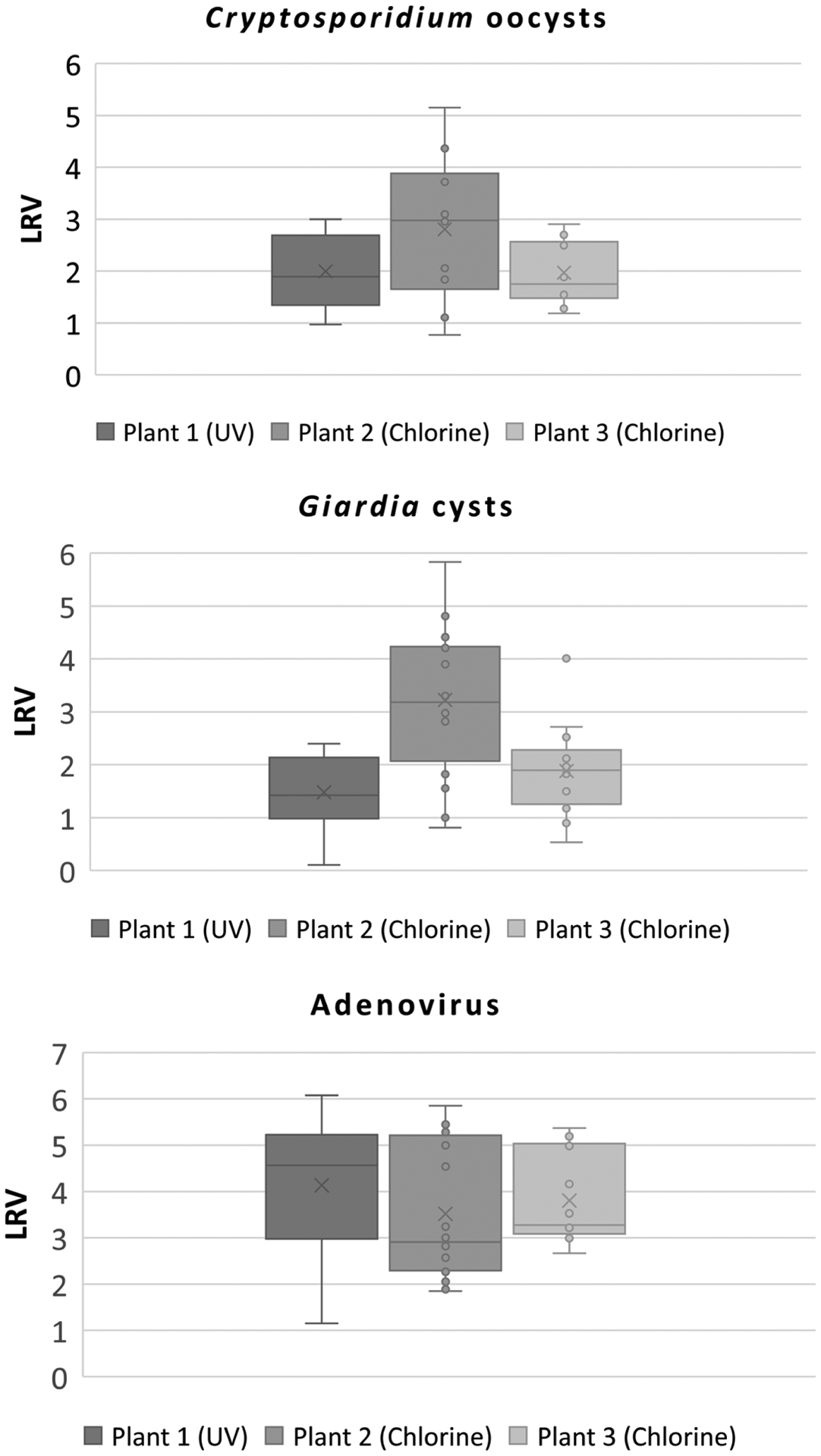
Log_10_ reduction values (LRVs) of protozoan parasites and infectious human adenovirus through wastewater treatment processes. Upper and lower whiskers denote the maximum and minimum values, respectively, and the median, 25th, and 75th quartiles are marked. Open circles and cross represent individual sample LRVs and the mean, respectively. The top left panel represents *Cryptosporidium* oocyst, the top right panel represents Giardia cyst, and bottom panel represents adenovirus LRVs.

**Figure 2.**
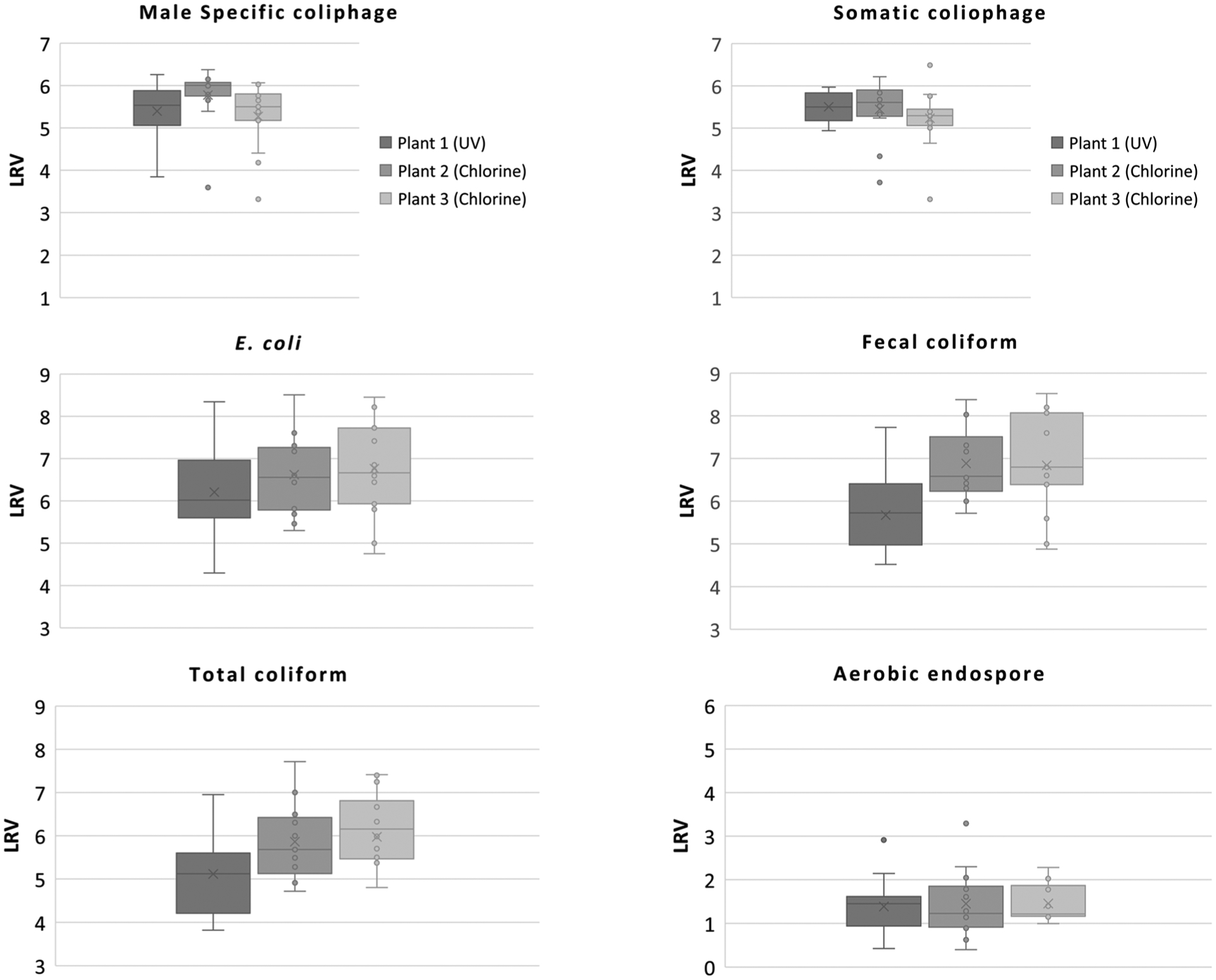
Log_10_ reduction values (LRVs) of microbial indicators through wastewater treatment processes. Upper and lower whiskers denote the maximum and minimum values, respectively, and the median, 25th, and 75th quartiles are marked. Open circles and cross represent individual sample LRVs and the mean, respectively. The top left panel represents male-specific coliphage, the top right panel represents somatic coliphage, the center left represents *E. coli*, the center right represents fecal coliform, the bottom left represents total coliform, and the bottom right panel represents aerobic endospore LRVs.

To determine treatment efficacies of the three treatment plants surveyed in this study, LRVs for the organisms evaluated were calculated. Box and whisker plot analyses were also performed, and statistical analyses (e.g., median and minimum-maximum values) are presented in Figures 1 and 2 and Tables S2–S4, respectively.

Compared to all the fecal indicator organisms tested except for aerobic endospores, relatively low median LRVs were observed. For the protozoan parasites, LRVs ranged from 1.75 to 2.98 and from 1.42 to 3.18 for *Cryptosporidium* oocysts and *Giardia* cysts, respectively (Figure 1 and Tables S3–S4). Cheng et al. [48] reported relatively low LRV with a range of 0.7–1.5 for *Cryptosporidium* spp. and 0.5–1.5 LRV for *Giardia* spp. during secondary treated processes without any disinfection (e.g., physical removal, primary settling, anaerobic- and/or aerobic-activated sludge biological treatment, and secondary clarification or settling.). Taran-Benshoshan et al. [42] reported a maximum LRV of 3.3 for *Giardia* cysts, which is consistent with the present study. The procedure used to detect these protozoa (i.e., IMS-IFA) does not determine viability, and thus, no information about their infectious potential can be ascertained. It is possible that this study underestimated the inactivation rates of both protozoan parasites because of limitations of the method. Moreover, many studies have reported that (oo)cysts are extremely resistant to chlorination [49–51], while relatively susceptible to UV disinfection [52–55]. It is thus fair to assume that Plant 1, which uses UV, might have lower levels of infectious (oo)cysts in effluent samples as compared with Plants 2 and 3, resulting in greater inactivation efficacy. Besides the infectious status of (oo)cysts, their speciation is another significant parameter to be considered for a more accurate estimation of microbial risks [18,56]. Not all species of *Cryptosporidium* spp. and *Giardia* spp. can infect humans. It is well documented, however, that two species of *Cryptosporidium* (e.g., *C. hominis* and *C. parvum*) and *Giardia duodenalis* are responsible for much of the waterborne disease outbreaks worldwide [57,58]. In the present study, it is unlikely all the *Cryptosporidium* oocysts and *Giardia* cysts detected are pathogenic to humans, since the IMS-IFA method used cannot distinguish the animal vs. zoonotic species of the parasite. Further characterization of protozoan parasites is needed for a more accurate microbial risk assessment to determine the public health significance associated with the sources of potable water.

Secondary treatment can achieve between 0.9 and 3.2 LRV for adenovirus [59]. Additionally, compared to protozoan parasites, viruses including adenovirus are more susceptible to chlorination. According to the California guideline for DPR treatment, Log reduction credits of viruses for conventional activated sludge secondary treatment with chlorination and low-dose UV are 5.9 and 2.4, respectively [60]. In the present study, the median LRVs for human adenovirus ranged from 2.91 to 4.57 for all three utilities (Figure 1 and Table S3). These values were several orders of magnitude lower than those of all the microbial indicators tested except for aerobic endospores. Many studies have reported that adenovirus is the most resistant to UV radiation [33,34,61–63]. Its high resistance to UV disinfection is one possible explanation for the high levels of infectious adenovirus detected in treated effluents from Plant 1. Because of this charcteristic, adenovirus has been utilized as a performance indicator for the direct evaluation of UV disinfection systems as components of water treatment processes, specifying a UV dose of 186 mJ/cm^2^ to achieve its 4-Log_10_ inactivation [64]. Further study on systematic microbial monitoring is needed to elucidate the efficacy of UV only and advanced oxidation with UV in full-scale water treatment plants. As shown in Figure 2, bacteriophage LRVs were around 5-Log_10_ for both male-specific and somatic bacteriophages regardless of the treatment used (chlorine vs. UV). The LRVs for bacteriophages were greater than those of human adenovirus, suggesting that male-specific and somatic bacteriophages are not appropriate candidates for viral indicators in DPR treatment. Fecal coliforms, *E. coli*, total coliforms, and aerobic endospores were also measured throughout the study. Unlike the pathogen levels noted above, the fecal indicator organism concentrations were orders of magnitude higher (Table 4), providing a broader dynamic range to assess the LRVs with at least 4-Log_10_ reduction sensitivity (Figure 2). Care should be taken, however, when using indicator datasets to assess treatment effects, since the values do not always correlate with the presence of pathogens, nor do they reveal actual treatment efficacies for those pathogens.

### 3.5. Engineering Implications for DPR on Microbial Quality

Overall, the results indicate a marked reduction in concentrations of microbial pathogens through the various treatment trains up to five LRVs. Despite the types of treatments used, detectable levels of (oo)cysts and human adenoviruses were observed in the effluents from all three plants (See min-max range in Table 2). Although there are still no regulations for the concentrations of protozoan parasites and enteric viruses in potable supplies, a minimum treatment level of 99.9% to 99.99% removal (3- to 4-log reduction) of these pathogens is required for drinking water in the U.S. [49]. UV light has been widely used in wastewater and reclaimed water treatment plants because of its effective inactivation efficacy against most waterborne pathogens, especially on *Cryptosporidium*, which are extremely resistant to chlorination. Regarding the microbial and disinfection by-product (MDBP) rules by USEPA [65,66], UV light outcompetes other chemical disinfectants to achieve effective treatment of a wide range of waterborne pathogens while producing negligible disinfection by-products [56,64,67].

Given that significant microbial reduction or inactivation of various microorganisms tested was observed during the wastewater treatment processes, this study provided additional insights on the prevalence, microbial densities, and treatment efficacies in three wastewater treatment plants that are considering the use of treated wastewater for DPR. However, notable differences in the concentrations of fecal indicator organisms and pathogens along with different microbial reduction values were found. Treatment technologies need to be evaluated by intensive monitoring for pathogens and fecal indicator organisms when considering DPR. Although three treatment plants were evaluated in this study and two disinfection approaches (e.g., chlorine vs. UV) were used, additional studies and a more intense sampling protocol are needed to compare treatment efficacies among the three plants via more rigorous statistical analyses.

## 4. Conclusions

We attempted to collect raw and secondary treated wastewater samples to demonstrate synoptic microbial loads as source water for DPR. Based on the fecal indicator and bacteriophage dataset, which assessed the viability of the organisms, results revealed the UV treatment efficacies. Moreover, it is well documented that UV light effectively inactivates protozoan parasites, which are extremely resistant to chlorination. Regarding the MDBP rules, UV may be a better treatment option than chlorine. More importantly, great care should be taken with making generalizations via deterministic approaches on the concentrations, occurrences, and prevalence of pathogens in the wastewater along with treatment efficacies of the entire wastewater treatment processes. Instead, a thorough characterization of the microbial composition of the wastewater and treatment process efficacies of the wastewater treatment plant considering DPR via stochastic models should be conducted. Additional research efforts are also needed to further understand treatment efficacies of traditional and advanced treatment processes that will be considered for treating water for direct or indirect reuse. This study will further aid in building a richer microbial occurrence database that can be used towards evaluating reuse guidelines and disinfection practices for water reuse practices. Additional studies focusing on occurrence, treatment efficacies, and development of QMRA models will be important to state/federal policy makers for DPR consideration and implementation.

## Supplementary Material

Supplementary Material Table S1. Sample volume collected (SVC), equivalent sample volume analyzed (ESVA), and detection limit (DL) for each testing microorganism.
Table S2. Median Log10 reduction values of protozoan parasites through the three wastewater treatment trains.
Table S3. Median Log10 inactivation values of human adenovirus and bacteriophages through the three wastewater treatment trains.
Table S4. Median Log10 inactivation values of fecal indicator organisms through the three wastewater treatment trains.
Figure S1. Overview of sampling schedule and microbial parameters measured.
Figure S2. Cryptosporidium oocyst concentrations in influent (blue) and effluent (red) wastewater samples.
Figure S3. Giardia cyst concentrations in influent (blue) and effluent (red) wastewater samples.
Figure S4. Log10 concentrations of infectious human adenovirus in influent (blue) and effluent (red) wastewater samples.
Figure S5. Log_10_ concentrations of male-specific and somatic coliphages in influent (blue) and effluent (red) wastewater samples.

## Figures and Tables

**Table 1.** Wastewater treatment plant (WWTP) characteristics.

WWTP	Customer Base	Processing Size (m^3^/Day)	Secondary Treatment Processes *	Disinfection Type
Plant 1	180,000	41.6 thousand	Aeration, Disc filter microscreens	Ultraviolet
Plant 2	28,000	9.1 thousand	Aeration, Trickling filters	Chlorine
Plant 3	20,000	8.0 thousand	Aeration	Chlorine

* Treatment processes include primary treatment (bar screens, grit chamber, primary clarification) and secondary treatment (activated sludge basin, secondary clarification).

**Table 2.** Prevalence and levels of human adenovirus, *Cryptosporidium*, and *Giardia*.

	Plant	Adenovirus (MPN/L)		*Cryptosporidium* (Oocysts/L)		*Giardia* (Cysts/L)	
		Influent-Grab	Effluent-HFUF ^a^	Influent-Grab	Effluent-HFUF ^a^	Influent-Grab	Effluent-HFUF ^a^
1	Prevalence	16/16 (100%)	9/16 (56%)	10/16 (63%)	13/16 (81%)	16/16 (100%)	16/16 (100%)
	Median [min-max]	3025 [8.64–65,480]	0.07 [0.05–511]	30.0 [10.0–190]	0.34 [0.11–8.55]	270 [50.0–2840]	10.0 [0.72–80.0]
2	Prevalence	16/16 (100%)	13/16 (81%)	11/16 (69%)	12/16 (75%)	16/16 (100%)	14/16 (88%)
	Median [min-max]	64.1 [3.17–14,878]	0.07 [0.02–0.54]	1150 [10.0–3420]	0.17 [0.02–49.8]	2400 [20.0–35,000]	1.45 [0.01–52.1]
3	Prevalence	14/15 (93%)	6/15 (40%)	10/15 (67%)	14/15 (93%)	14/15 (93%)	15/15 (100%)
	Median [min-max]	25.9 [10.23–5797]	0.02 [0.02–0.04]	15.0 [8.00–150]	0.23 [0.01–1.42]	717 [80.0–3140]	4.74 [0.10–77.0]

a Equivalent sample volume analyzed (ESVA) for adenovirus and parasites were 50 L and 10–100 L, respectively. For example, 50 L was calculated using the following equation. ESVA = (200 mL/400 mL UF eluate) × 100 L of effluent sample filtered. The detection limits (DL) of grab samples and effluent-UF for adenovirus were 2.5 and 0.02 MPN/mL, respectively.

**Table 3.** Prevalence and levels of male-specific and somatic bacteriophages.

	Plant	Male Specific (PFU/mL)			Somatic (PFU/mL)		
		Influent-Grab	Effluent-Grab	Effluent-HFUF ^a^	Influent-Grab	Effluent-Grab	Effluent-HFUF ^a^
1	Prevalence	16/16 (100%)	0/16 (0%)	3/16 (19%)	16/16 (100%)	0/16 (0%)	5/16 (31%)
	Median [min-max]	1433 [52–7400]	ND	0.004, 0.005, 0.043	1458 [355–3850]	ND	0.005 [0.004–0.009]
2	Prevalence	14/14 (100%)	1/14 (7%)	1/14 (7%)	13/13 (100%)	1/14 (7%)	2/14 (14%)
	Median [min-max]	4700 [20–11,700]	100	0.038	1865 [110–7300]	2	0.004, 0.25
3	Prevalence	15/15 (100%)	1/15 (6.7%)	1/15 (6.7%)	15/15 (100%)	0/15 (0%)	2/15 (13.3%)
	Median [min-max]	1555 [52–6050]	100	1.11	985 [225–15, 150]	ND	0.005, 1.01

a Equivalent sample volume analyzed (ESVA) was 250 mL, which was calculated using the following equation. ESVA = (1 mL/400 mL UF eluate) × 100 L of effluent sample filtered. The detection limits (DLs) of grab samples and effluent-UF were 1 and 0.004 PFU/mL, respectively. ND: not determined.

**Table 4.** Prevalence and Log_10_ concentrations of bacterial indicators.

Plant [Log_10_ CFU/100 mL]		*E. coli*		Fecal Coliform		Total Coliform		Endospore	
		Influent	Effluent	Influent	Effluent	Influent	Effluent	Influent	Effluent
1	Prevalence	16/16 (100%)	16/16 (100%)	16/16 (100%)	16/16 (100%)	16/16 (100%)	16/16 (100%)	16/16 (100%)	16/16 (100%)
	Median [min-max]	6.99 [5.80–8.84]	0.96 [0.30–1.68]	7.30 [5.30–9.38]	1.69 [0.99–2.81]	8.01 [6.92–9.38]	2.91 [2.31–3.38]	4.34 [3.30–5.35]	2.96 [2.31–3.38]
2	Prevalence	14/14 (100%)	7/14 (50%)	14/14 (100%)	10/14 (71%)	14/14 (100%)	12/14 (86%)	14/14 (100%)	14/14 (100%)
	Median [min-max]	6.94 [5.30–8.81]	0.61 [0.30–2.39]	7.37 [6.30–8.94]	0.76 [0.30–2.04]	8.16 [6.99–9.38]	2.77 [0.49–3.38]	4.20 [2.70–5.47]	2.82 [1.60–4.30]
3	Prevalence	15/15 (100%)	2/15 (13%)	15/15 (100%)	5/15 (33%)	15/15 (100%)	14/15 (93%)	15/15 (100%)	15/15 (100%)
	Median [min-max]	6.72 [5.00–8.45]	0.30, 2.29	6.93 [5.00–8.94]	0.61 [0.30–2.38]	7.51 [5.30–9.38]	1.83 [0.80–3.19]	4.42 [3.79–5.48]	2.93 [2.40–3.99]

Note: One hundred milliliters of influent and effluent grab samples were analyzed (i.e., equivalent sample volume analyzed (ESVA) was 100 mL that corresponds to a detection limit (DL) of 1 CFU/100 mL).

## Data Availability

The data presented in this study are available on request from the corresponding author. The data are not publicly available anywhere except the EPA network drive.
