# Supplementary material for: Understanding Microbial Loads in Wastewater Treatment Works as Source Water for Water Reuse
Source: Water (Basel). Author manuscript; Available in PMC 2022 May 21. (PMC8597597; doi:10.3390/w13111452)
Supplement: Supplementary Material — Table S1. Sample volume collected (SVC), equivalent sample volume analyzed (ESVA), and detection limit (DL) for each testing microorganism. Table S2. Median Log10 reduction values of protozoan parasites through the three wastewater treatment trains. Table S3. Median Log10 inactivation values of human adenovirus and bacteriophages through the three wastewater treatment trains. Table S4. Median Log10 inactivation values of fecal indicator organisms through the three wastewater treatment trains. Figure S1. Overview of sampling schedule and microbial parameters measured. Figure S2. Cryptosporidium oocyst concentrations in influent (blue) and effluent (red) wastewater samples. Figure S3. Giardia cyst concentrations in influent (blue) and effluent (red) wastewater samples. Figure S4. Log10 concentrations of infectious human adenovirus in influent (blue) and effluent (red) wastewater samples. Figure S5. Log10 concentrations of male-specific and somatic coliphages in influent (blue) and effluent (red) wastewater samples. [file NIHMS1749622-supplement-Supplementary_Material.pdf]

water

Article

Understanding Microbial Loads in Wastewater Treatment Works as Source Water for Water Reuse

Hodon Ryu [1], Yao Addor [2], Nichole E. Brinkman [1], Michael W. Ware [2], Laura Boczek [1], Jill Hoelle [1], Jatin H. Mistry [3], Scott P. Keely [2] and Eric N. Villegas [2,*]

1 United States Environmental Protection Agency, Office of Research and Development, Center for Environmental Solutions and Emergency Response, Cincinnati, OH 45268, USA; ryu.hodon@epa.gov (H.R.); brinkman.nichole@epa.gov (N.E.B.); boczek.laura@epa.gov (L.B.); hoelle.jill@epa.gov (J.H.)
2 United States Environmental Protection Agency, Office of Research and Development, Center for Environmental Measurement and Modeling, Cincinnati, OH 45268, USA; addorys@mail.uc.edu (Y.A.); ware.michael@epa.gov (M.W.W.); keely.scott@epa.gov (S.P.K.)
3 United States Environmental Protection Agency, Region 6, Dallas, TX 75270, USA; mistry.jatin@epa.gov
* Correspondence: villegas.eric@epa.gov

Citation: Ryu, H.; Addor, Y.; Brinkman, N.E.; Ware, M.W.; Boczek, L.; Hoelle, J.; Mistry, J.H.; Keely, S.P.; Villegas, E.N. Understanding Microbial Loads in Wastewater Treatment Works as Source Water for Water Reuse. *Water* **2021**, *13*, 1452. https://doi.org/10.3390/w13111452

Academic Editor: Andreas N. Angelakis

Received: 16 April 2021
Accepted: 19 May 2021
Published: date

Publisher's Note: MDPI stays neutral with regard to jurisdictional claims in published maps and institutional affiliations.

Copyright: © 2021 by the authors. Submitted for possible open access publication under the terms and conditions of the Creative Commons Attribution (CC BY) license (https://creativecommons.org/licenses/by/4.0/).

Table S1. Sample volume collected (SVC), equivalent sample volume analyzed (ESVA)**, and detection limit (DL)** for each testing microorganism.

Sampling Site*	Sample Type	Sample Volume (SVC)	Parasites		Adenovirus		Bacteriophages		Bacterial Indicators	
			ESVA	DL ((oo)cysts/L)	ESVA	DL (MPN/L)	ESVA	DL (PFU/mL)	ESVA	DL (CFU/100 mL)
Influent	Grab	1 L	100–125 mL	8–10	400 mL	2.5	1 mL	1	100 mL	1
	HFUF	100 L	ND	ND	ND	ND	ND	ND	ND	ND
Effluent	Grab	1 L	ND	ND	ND	ND	1 mL	1	100 mL	1
	HFUF	100 L	10–100 L	0.01–0.1	50 L	0.02	250 mL	0.004	ND	ND

*Influent samples: primary treated wastewater (e.g., primary clarified/settled), Effluent samples: final treated wastewater (e.g., secondary treatment and disinfection). **Calculations were described in Tables 2-4. ND: not determined.

Table S2. Median Log_{10} reduction values of protozoan parasites through the three wastewater treatment trains. Minimum-Maximum Log_{10} values are in parenthesis.

Log Reduction [Log_{10} (Inf/eff)]	*Cryptosporidium* Oocysts	*Giardia* Cysts
Plant 1 (UV)	1.89 (0.97–3.00)	1.42 (0.11–2.40)
Plant 2 (Chlorine)	2.98 (0.77–5.15)	3.18 (0.81–5.83)
Plant 3 (Chlorine)	1.75 (1.19–2.90)	1.90 (0.53–4.01)

Table S3. Median Log_{10} inactivation values of human adenovirus and bacteriophages through the three wastewater treatment trains. Minimum-Maximum Log_{10} values are in parenthesis.

Log Inactivation [Log_{10} (Inf/eff)]	Male Specific Bacteriophage	Somatic Bacteriophage	Human Adenovirus
Plant 1 (UV)	5.54 (3.85–6.26)	5.50 (4.94–5.97)	4.57 (1.15–6.08)
Plant 2 (Chlorine)	6.01 (3.59–6.38)	5.61 (4.33–6.21)	2.91 (1.85–5.85)
Plant 3 (Chlorine)	5.50 (3.32–6.07)	5.29 (3.32–6.49)	3.27 (2.66–5.37)

Table S4. Median Log_{10} inactivation values of fecal indicator organisms through the three wastewater treatment trains. Minimum-Maximum Log_{10} values are in parenthesis.

Log Inactivation [Log_{10} (Influent/effluent)]	*E. coli*	Fecal Coliforms	Total Coliforms	Aerobic Endospores
Plant 1 (UV)	6.02 (4.30–8.35)	5.73 (2.79–7.73)	5.12 (3.82–6.95)	1.45 (0.43–2.91)
Plant 2 (Chlorine)	6.56 (5.30–8.51)	6.58 (5.72–8.38)	5.68 (4.72–7.72)	1.23 (0.40–3.29)
Plant 3 (Chlorine)	6.66 (4.75–8.45)	6.80 (4.88–8.52)	6.16 (2.76–7.42)	1.21 (0.99–2.28)

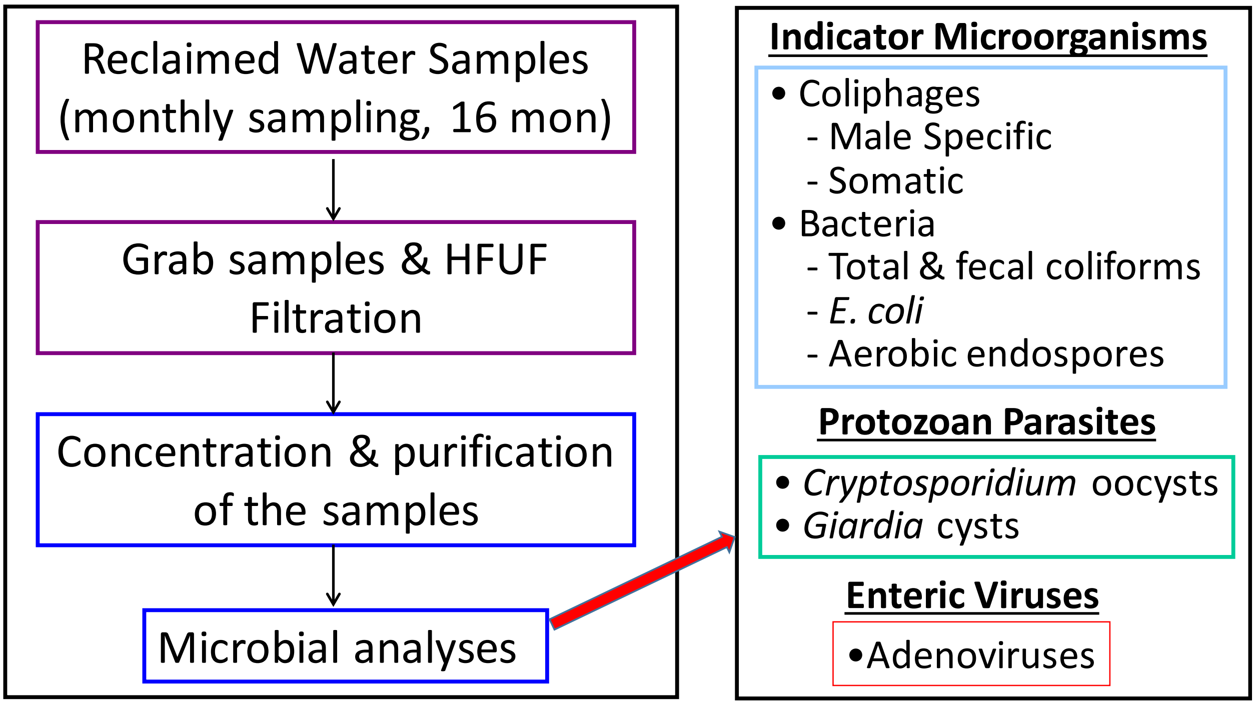

Figure S1. Overview of sampling schedule and microbial parameters measured.

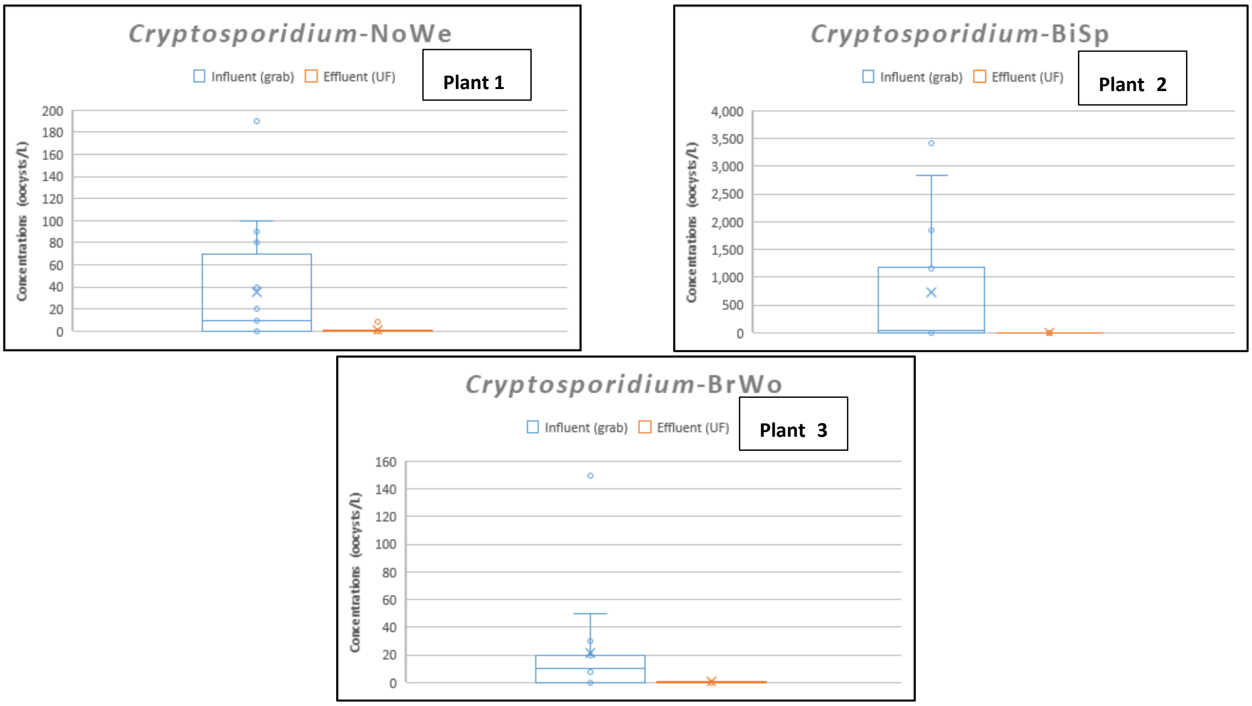

Figure S2. *Cryptosporidium* oocyst concentrations in influent (blue) and effluent (red) wastewater samples.

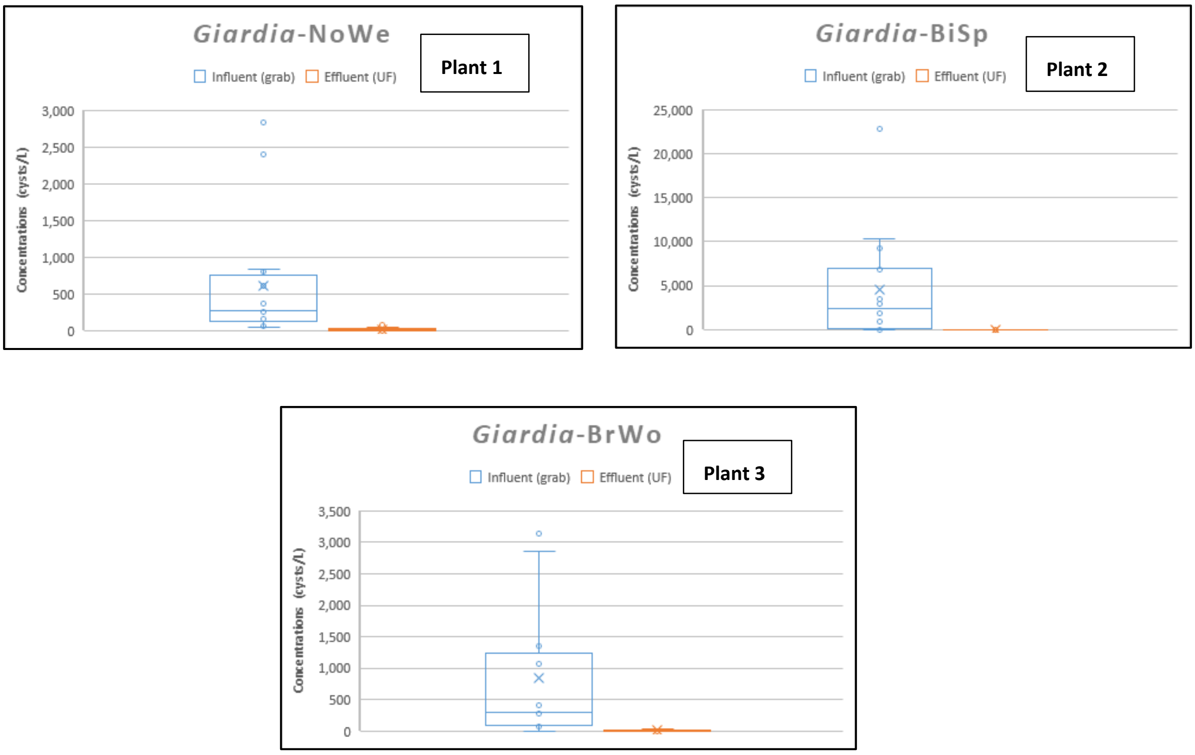

Figure S3. *Giardia* cyst concentrations in influent (blue) and effluent (red) wastewater samples.

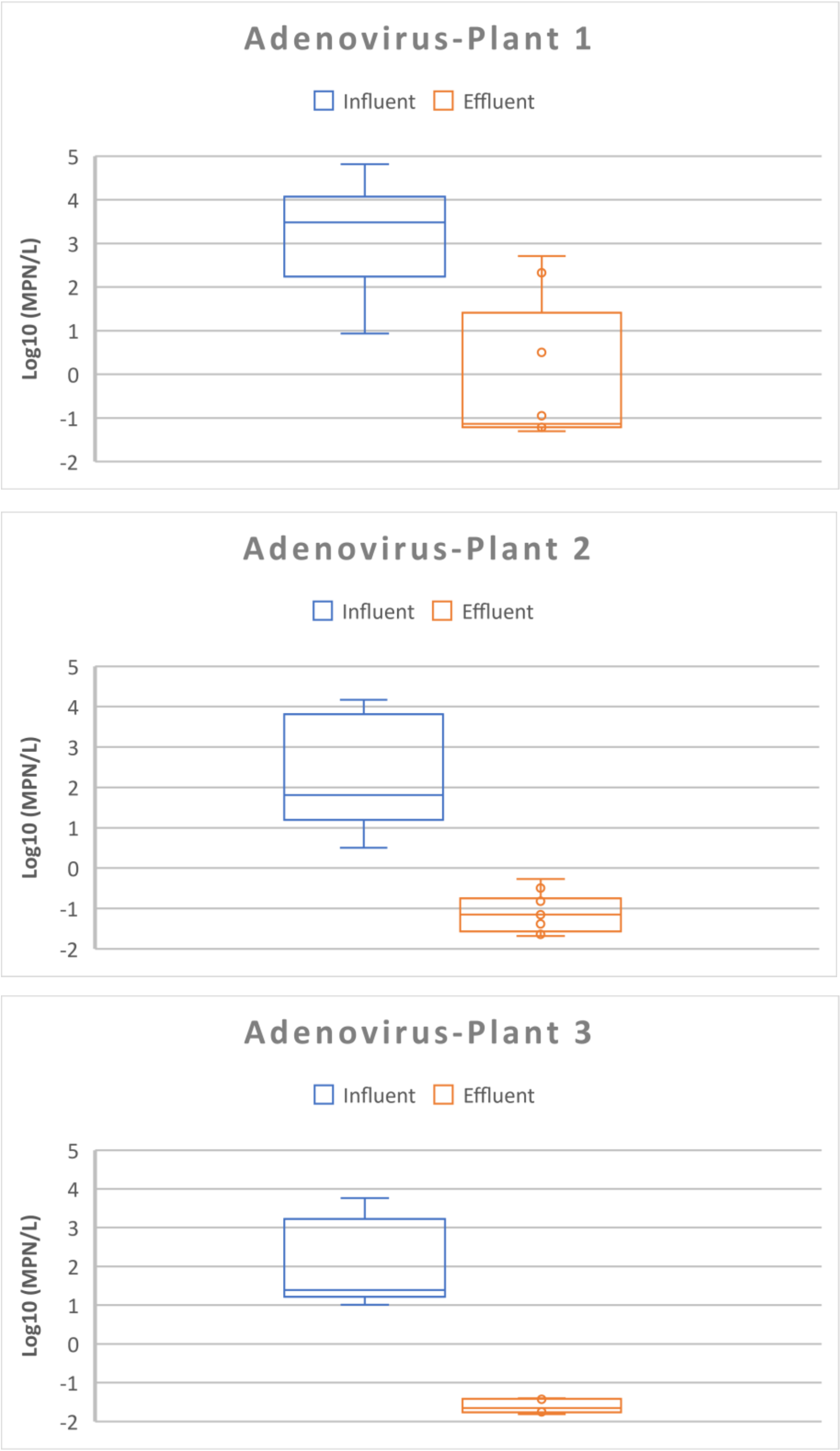

Figure S4. Log_{10} concentrations of infectious human adenovirus in influent (blue) and effluent (red) wastewater samples.

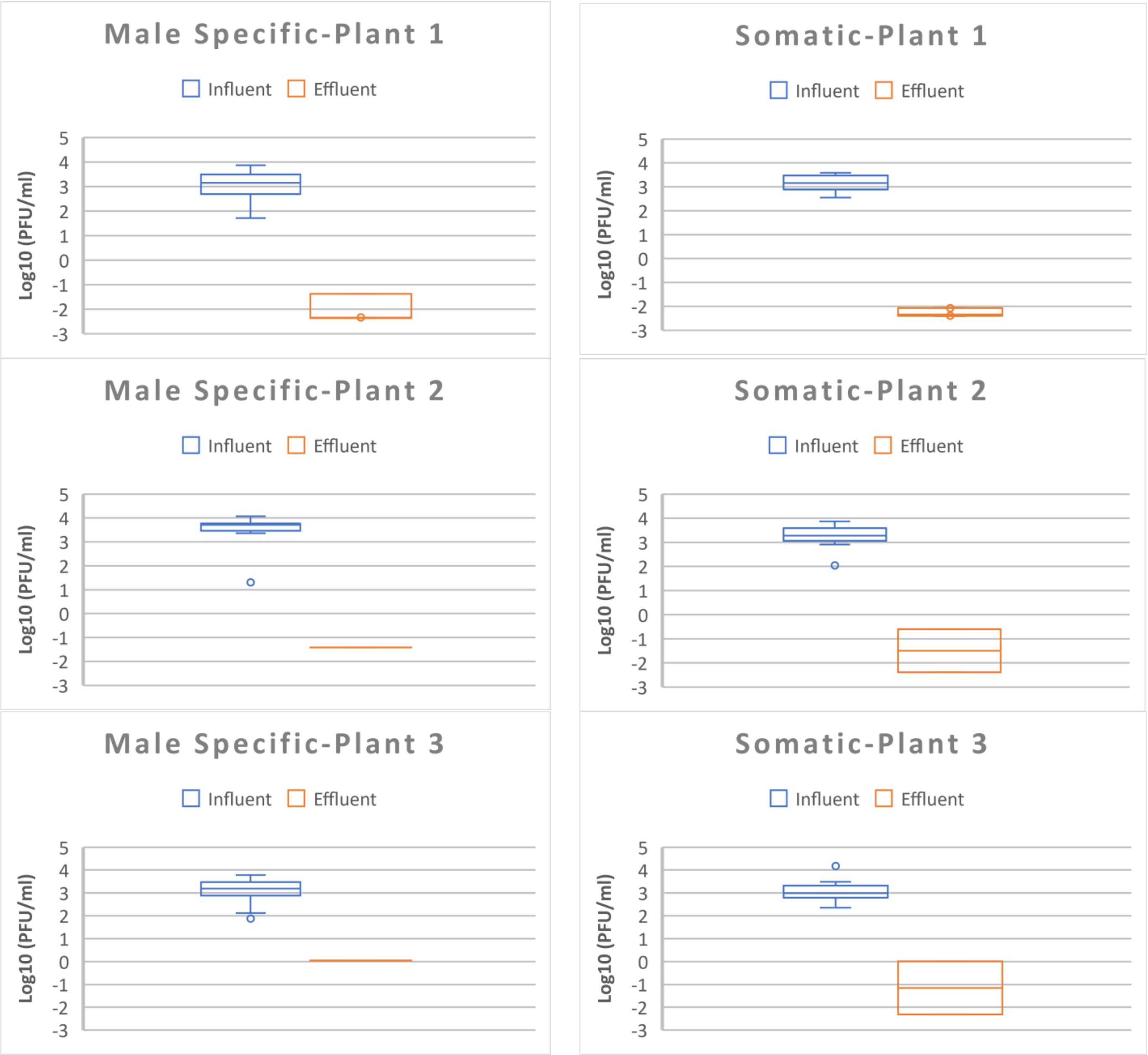

Figure S5. Log_{10} concentrations of male specific and somatic coliphages in influent (blue) and effluent (red) wastewater samples.
